# The blast wounded of Raqqa, Syria: observational results from an MSF-supported district hospital

**DOI:** 10.1186/s13031-019-0214-0

**Published:** 2019-06-20

**Authors:** Jennifer OKeeffe, Larissa Vernier, Vanessa Cramond, Shazeer Majeed, Antonio Isidro Carrion Martin, Maartje Hoetjes, Mohana Amirtharajah

**Affiliations:** 1 Médecins Sans Frontières, Tal-Abyad, Syria; 2 Médecins sans Frontières, Naritaweg 10, 1043 BX Amsterdam, the Netherlands; 3 Médecins sans Frontières, Lower Ground Floor, Chancery Exchange, 10 Furnival Street, London, EC4A 1AB UK

**Keywords:** Blast, Syria, Trauma, Conflict settings, Surgery, Wound care

## Abstract

**Background:**

In June 2017, the U.S.-backed Syrian Democratic Forces (SDF) launched a military operation to retake the city of Raqqa, Syria, from the so-called Islamic State. The city population incurred mass numbers of wounded. In the post-offensive period, the population returned to a city (Raqqa) contaminated with improvised explosive devices (IEDs) and explosive remnants of war (ERWs), resulting in a second wave of wounded patients. Médecins Sans Frontières (MSF) supported a hospital in Tal-Abyad (north of Raqqa) and scaled up operations in response to this crisis. We describe the cohort of blast-wounded cases admitted to this hospital in order help prepare future humanitarian responses.

**Methods:**

We retrospectively extracted data from clinical charts in the MSF-supported hospital. We included all new admissions for blast-wounded patients with key data elements documented. We performed comparative analyses from the offensive period (June 6, 2017 to October 17, 2017) and the post-offensive period (October 18, 2017 to March 17, 2018).

**Results:**

We included 322 blast related injuries. There were more than twice the number of cases with blast injuries in the post-offensive period as the offensive period (225 vs. 97, *p =* <.001). The offensive period saw a significantly higher proportion of female patients (32.0%, *n* = 31 vs. 11.1%, *n* = 25, *p* < 0.001) and paediatric patients (42.3%, *n* = 41 vs 24.9%, *n* = 56, *p* = 0.002). Blast-injured patients in the post-offensive period included more cases with multiple traumatic injuries (65.8%, *n* = 148 vs. 39.2%, *n* = 38, *p* < 0.001). The treatment of the blast-injured cases in the post-offensive period was more labor intensive with those patients having a higher median number of interventions (2 vs 1, *p* = <0.001) and higher median number of days in hospital (7 vs 4, *p* = < 0.001).

**Conclusions:**

In the wake of the Raqqa offensive, the MSF-supported district hospital received an unpredicted second, larger and more complex wave of blast-wounded cases as the population returned to a city strewn with IEDs and ERWs. These findings indicate the high risk of traumatic injury to the population even after warring factions have vacated conflict zones. Medical humanitarian actors should be prepared for a continued and scaled up response in areas known to be highly contaminated with explosive ordnance.

## Background

In 2014, in the midst of the war raging in Syria, the so-called Islamic State of Iraq and the Levant (ISIL) seized control of large areas of western Syria and eastern Iraq, setting up the city of Raqqa in northern Syria as their de facto capital. In response, the Kurdish dominated Syrian Democratic Forces (SDF) aligned with the United States (US) led coalition of forces launched Operation Wrath of Euphrates [1], an operation aimed at taking the city of Raqqa as part of a larger campaign to drive ISIL out of the region. The last phase of the operation, the Battle of Raqqa military offensive, began on June 6, 2017 and officially ended on October 17, 2017 [2]. SDF ground troops supported by heavy US airstrikes encircled the city, effectively trapping combatants and civilians alike inside. The city population incurred a large number of wounded attributed to airstrikes. Monitoring groups put the total number of civilian casualties between 1300 and 1800 as a result of nearly 4500 airstrikes and the use of over 20,000 munitions between the months of May and October, 2017 [3, 4].

When active combat ended, the civilian population began to return to Raqqa, a city was strewn with improvised explosive devices (IEDs) and explosive remnants of war (ERWs) [5]. ISIL has been known to leave booby traps when evacuating an area previously under their control, but ERWs, including bombs and missiles left by other armed forces, were also present in the area [6–8]. In the months following the coalition offensive, 658 injuries and 130 deaths were reported from IEDs, ERWs, booby traps and other unexploded ordnance (UXO) in the city^1^[9]. The actual number is likely much higher due to the unreported deaths that occurred before people could reach medical assistance. In Raqqa, high political and security concerns, compounded by the number and magnitude of explosives used in the offensive and post-offensive periods, made humanitarian access difficult. Many humanitarian health actors were unable or unwilling to access the city of Raqqa in the months following the military offensive; thus, information about the humanitarian situation in Raqqa during this time remains extremely limited.

Médecins Sans Frontières (MSF) is an international, independent, humanitarian medical organisation that provides medical assistance to vulnerable populations, including those affected by conflict. MSF-OCA (Operational Center Amsterdam) began supporting a district hospital in Tal Abyad, north of Raqqa town, in 2016, mainly in the form of medical supplies. In June 2017, it scaled up its support, adding staff incentives, technical assistance and international staff to care for the heavy caseload of traumatic injuries resulting from the military offensive. Tal Abyad is located 90 km north of Raqqa, posing a minimum of 120 min transport time without delays. In the context of the offensive period, checkpoints, combat conditions and other barriers often meant the time for transport was much longer. In Raqqa governate, MSF operated alongside and coordinated with a presence of military and non-military health actors. It served as the primary referral facility for acute injuries and illness for most of the civilian population.

After the initial combat and airstrikes ended, there was a respite in the number of casualties seen, and MSF prepared to transition its support away from trauma care. However, in late October, as the population unexpectedly returned to the city prior to decontamination, the hospital began to see an increase in blast admissions. Therefore, MSF scaled up its trauma-related operations, adding a trauma stabilization point (TSP) within the city limits to respond to the number and complexity of blast injuries.

Although recent work has described the burden of injury to civilian populations during active fighting in a similar context, these studies do not provide an indication of the burden of trauma that can occur during the immediate post-offensive period in a highly ordnance-contaminated urban area [10, 11]. The objective of this study was therefore to describe the cohort of blast-wounded cases admitted to the MSF supported district hospital during the Raqqa military offensive and the first months of the post-offensive period in order to better prepare existing and future humanitarian responses.

## Methods

### Study design

This study used a retrospective observational descriptive design. It drew on routinely collected programme data from an MSF supported district hospital in northern Syria.

### Study period and setting

This study considers the period from June 6, 2017 to October 17, 2017 as the Raqqa offensive period. The post-offensive period began October 18, 2017 and ended on March 17, 2018 when the blast caseload had begun to decrease. The MSF supported district hospital was located in Tal Abyad, northern Syria and was the only functioning public hospital in Raqqa Governorate throughout the Raqqa offensive and post-offensive periods. In this capacity, it served as the primary trauma referral site for all medical actors, delivering acute surgical care to severely injured patients.

### Study population

The study population consisted of all new blast-wounded admissions to the MSF supported district hospital in the offensive or post offensive period with all key data elements documented in their medical chart. Blast-wounded were defined as patients with physical trauma from direct or indirect exposure to an explosion including improvised explosive devices (IEDs), unexploded ordnance (UXO), mines, missiles, projectiles and fragments. New admissions were defined as patients who were admitted to the hospital for the first time for one set of wounds.

### Data collection and analysis

The study team reviewed all available medical charts for patients admitted to the MSF supported district hospital during the offensive or post-offensive period and extracted key data elements using a data extraction paper form. Data were entered into an online database using Kobo Toolbox and exported to Stata version 14.2 for analysis. The data variables collected for analysis included patient sex, age, admission and exit dates, classification and location of injuries, presence of multiple injuries, exit status, number and type of intervention, anaesthesia type, surgical complications (including patient infections) and anaesthesia complications. Patients were considered to have multiple injuries if they had more than one documented classification of injury or one documented classification of injury in more than one location on the body. Interventions included any procedure performed in the operating theatre by a surgeon including formal surgeries, wound care and all procedures requiring any type of anaesthesia.

Analyses compared results in the offensive and post-offensive periods to determine differences by specified variables. Variables were tested for normality using skewness-kurtosis normality tests due to the large number of tied data. None of the variables tested passed normality tests and nonparametric tests were used for comparisons. The team performed chi square and Fisher’s exact tests to test differences in blast injuries by time period and sex, exit status and presence of multiple injuries. Chi square tests were one tailed with one degree of freedom and α = 0.5. The team used Wilcoxon rank sum tests to measure differences by time period and age, number of interventions, length of stay, and time between admission and first intervention. Findings were considered significant at a *p* value of < 0.05.

### Ethical approval

The study fulfilled the exemption criteria set by the Médecins Sans Frontières Ethics Review Board for a posteriori analyses of routinely collected clinical data and was conducted with permission from the MSF-OCA Medical Director. No personal identifiers were included in databases or used for analyses. Data sets were password protected and only accessible to the study team.

## Results

**Fig. 1:**
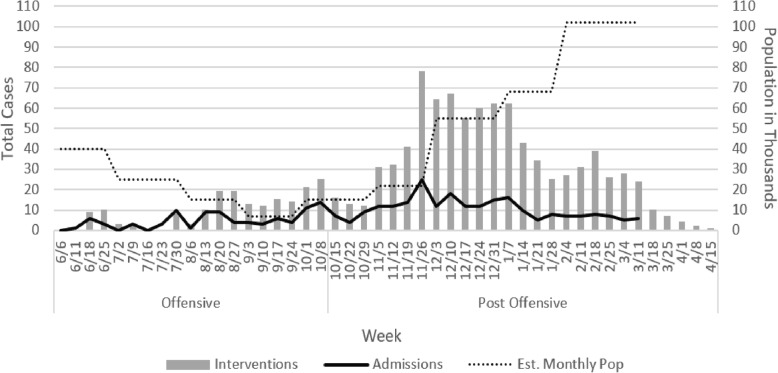
MSF district hospital new blast case admissions and interventions in the offensive and post-offensive period, Tal Abyad, Syria, June 2017–March 2087(Interventions in the post-offensive period include interventions on cases admitted to the district hospital in the offensive period. Admissions after March 18, 2018 were not included in analysis. Population estimates in the post-offensive period were difficult to find and were calculated based on estimate of population remaining at the end of the offensive combined with the estimated number of returnees during the month) [12]

Three hundred and twenty-two blast related admissions were included in the study, 97 from the offensive period and 225 from the post-offensive period (*p* = <.001). Figure 1 shows the evolution of admissions and interventions over the course of the study period. The 97 blast admissions made up 68.8% of the total weapon wounded admissions to the hospital during the offensive period. Data on the total war wounded admissions were not available in the post-offensive period. Table 1. describes the results of variables collected from patient data.

**Table 1:** Comparison of new blast admissions to the MSF district hospital in north Syria during the Raqqa military offensive and post-offensive periods, Tal Abyad, Syria, June 2017–March 2018

Variable (n; %)	Offensive	Post-offensive	Test Statistic	*p* value
Cases				
Total	97 (100.0)	225 (100.0)		
Median (IQR)	49 (48)	113 (113)	Z = -8.0	<0.001
Sex				
Female	31 (32.0)	25 (11.1)	χ^2^ = 20.50	<0.001
Male	66 (68.0)	200 (88.9)		
Age (years)				
Median (IQR)	20 (17)	27 (17)		
< 18	41 (42.3)	56 (24.9)	χ^2^ = 9.7	0.002
≥ 18	56 (57.7)	169 (75.1)		
Injuries per patient				
Median (IQR)	1 (1)	2 (1)		
Single	59 (60.8)	77 (34.2)	χ^2^ = 19.7	<0.001
Multiple	38 (39.2)	148 (65.8)		
Interventions per Patient				
Median (IQR)	1 (3)	2 (4)	Z = -4.0	<0.001
0	27 (27.8)	23 (10.2)		
1	30 (30.9)	64 (28.4)		
2–5	30 (28.9)	91 (40.4)		
≥6	10 (10.3)	47 (20.9)		
Length of Stay (days)				
Median (IQR)	4 (7)	7 (11)	Z = -3.7	<0.001
0	16 (16.5)	6 (2.7)		
1–7	52 (53.6)	116 (51.6)		
8–14	14 (14.4)	53 (23.6)		
≥15	15 (15.5)	50 (22.2)		
Admission/Intervention Interval (days)				
Median (IQR)	0 (1)	0 (1)	Z = 0.4	0.678
0	48 (68.6)	141 (69.8)		
1	11 (15.7)	42 (20.8)		
≥2	11 (15.7)	19 (9.4)		
Exit Status^*Ɨ^				
Discharged	79 (81.4)	164 (72.9)	–	0.047
Defaulted	8 (8.3)	40 (17.8)		
Transferred	6 (6.2)	18 (8.0)		
Died	4 (4.1)	3 (1.3)		
Patient Infections	8 (8.3)	26 (11.6)	χ^2^ = 0.785	0.375
Surgical Complications	17 (7.5)	40 (4.7)	χ^2^ = 2.865	0.091
Anaesthetic Complications^*^	3 (1.3)	6 (0.7)	χ^2^ = 0.841	0.287

*Fisher’s exact test used in place of chi square

^Ɨ^*p* value of <0.008 required for significance with Bonferroni correction (0.05/6)

The majority of blast wounded patients admitted for care during both time periods were adult males (82.6%, *n* = 266). However, the offensive period saw a significantly higher proportion of female patients (32.0%, *n* = 31 vs. 11.1%, *n* = 25, *p* < 0.001) and paediatric patients (42.3%, *n* = 41 vs 24.9%, *n* = 56, *p* = 0.002) than the post-offensive period. The trend in new admissions of paediatric patients changed over time. The first months of the offensive period, June and July, saw a high proportion of new paediatric admissions with 70.0% (*n* = 7) and 62.5% (*n* = 5) of all admissions in those months. By September, the proportion had dropped to 30% or below and remained that low until the end of the post-offensive period. In February and March, the hospital again began seeing more paediatric patients, documenting 36.7% (*n* = 11) and 40.0% (*n* = 6) as a proportion of all admissions during those months.

One hundred and forty-six injuries were documented in the 97 patients from the offensive period and 425 injuries were documented in the 225 patients from the post-offensive period. The occurrence of multiple traumatic injuries in a patient were more common during the post-offensive period compared to the offensive one (65.8%, *n* = 148 vs. 39.2%, *n* = 38, *p* < 0.001).

The types of injuries did not vary significantly between the two time periods. Table 2 describes the types of injuries received in the two periods. In the offensive and post-offensive periods, soft tissue injuries comprised the largest category with 45.2% (*n* = 66) and 40.5% (*n* = 172) respectively. This was followed by open fractures with 17.1% (*n* = 25) and 23.3% (*n* = 99), torso injuries with 15.1% (*n* = 22) and 9.2% (*n* = 39), and traumatic amputations with 10.3% (*n* = 15) and 11.5% (*n* = 49) during the offensive and post-offensive periods respectively. These four categories made up 87.7% of the offensive period injuries and 84.5% of the post-offensive period injuries. The other categories included in analysis were closed fractures, cranial injuries, vascular injuries, eye injuries, spinal injuries, degloving, burns and other injuries. Each made up 4.0% or less of the total injuries for both periods.

**Table 2:** New blast admissions to the MSF district hospital in north Syria during the Raqqa military offensive and post-offensive periods by injury, Tal Abyad, Syria, June 2017–March 2018

Variable	Offensive	Post-offensive
	n (%)	n (%)
Injury	146 (100.0)	425 (100.0)
Soft Tissue	66 (45.2)	172 (40.5)
Open Fracture	25 (17.1)	99 (23.3)
Torso Injury	22 (15.1)	39 (9.2)
Traumatic Amputation	15 (10.3)	49 (11.5)
Burn	4 (2.7)	10 (2.4)
Cranial	4 (2.7)	8 (1.9)
Vascular	3 (2.1)	16 (3.8)
Closed Fracture	2 (1.4)	13 (3.1)
Degloving	2 (1.4)	5 (1.2)
Other	2 (1.4)	3 (0.7)
Eye	1 (0.7)	11 (2.6)

Two hundred and twenty-six interventions were performed during the offensive period and 853 in the post-offensive period, described in Table 3. The post-offensive period had a higher median number of interventions per case (2) than the offensive period (1) (*p* < 0.001). The median length of stay in the hospital was also higher in the post-offensive period with 7 days compared to 4 days during the offensive period (p < 0.001). There was no difference in the interval between admission and first intervention with both time periods having a median of 0 days delay (*p* = 0.678). The surgical complication rate was 7.5% (*n* = 17) in the offensive and 4.7% (*n* = 40) in the post-offensive period (*p* = 0.091). The anaesthetic complication rate was 1.3% (*n* = 3) in the offensive period and 0.7% (*n* = 6) in the post-offensive period (*p* = 0.287).

**Table 3:** New blast admissions to the MSF district hospital in north Syria during the Raqqa military offensive and post-offensive periods by procedure

Variable	Offensive	Post-offensive
	n (%)	n (%)
Procedure	409 (100.0)	1560 (100.0)
Wound Care	342 (83.6)	1328 (85.1)
Orthopaedic	38 (9.3)	162 (10.4)
Laparotomy	15 (3.7)	43 (2.8)
Thoracic	7 (1.7)	5 (0.3)
Vascular	4 (1.0)	19 (1.2)
Other	3 (0.7)	3 (0.3)

Four hundred and nine procedures were performed during the 226 interventions in the offensive period and 1560 procedures during the 853 interventions in the post-offensive period. In both periods, wound care consisting of debridements, change of dressing, and wound closure comprised the vast majority of procedures. Surgeons at the hospital performed 168 debridements in the offensive period and 608 debridements in the post-offensive making up 41.1 and 39.0% of all procedures respectively. An additional 127 (31.1%) and 562 (36.0%) changes of dressing were performed and 32 (7.8%) and 102 (6.5%) wound closures. Orthopaedic procedures were the second largest category with 38 (9.3%) procedures in the offensive and 162 (10.4%) in the post-offensive periods. These were mainly primary placement of external fixation with 16 (3.9%) and 84 (5.4%) procedures and amputation with 17 (4.2%) and 48 (3.1%) procedures.

Patients were discharged when they were deemed to be medically stable, could have dressing changes as an outpatient and were either judged to be independent enough to perform routine daily tasks or had sufficient assistance at home for these tasks. In the offensive period, 79 (81.4%) patients were discharged compared to 164 patients (72.9%) in the post-offensive period. There were 8 (8.3%) patients who defaulted, including those discharged against medical advice, in the offensive period and 40 (17.8%) patients who defaulted in the post-offensive period. There were 6 (6.2%) transferred patients in the offensive period and 18 (8.0%) in the post-offensive period. The intra-hospital mortality rate among those admitted was 4.1% (*n* = 4) in the offensive period and 1.3% (*n* = 3) for the post-offensive period. The offensive period had a patient infection rate of 8.3% (*n* = 8) while the post-offensive period had a rate of 11.6% (*n* = 26) (*p* = 0.375).

## Discussion

This retrospective observational study described the cohort of blast-wounded cases admitted to the MSF district hospital of Tal Abyad during the Raqqa military offensive and the first months of the post-offensive period. These data show that despite the cessation of active fighting, the burden of injury from IED’s and ERW’s remained extremely high, causing a heavy second wave of war-wounded patients during the post-offensive period. At the time, MSF was ready to scale down its surgical response as the acute offensive period had ended. This second wave created a significant burden of work for the MSF supported hospital that was unanticipated and for which it was not prepared. The burden of blast injuries as a proportion to overall trauma in urban conflict settings has increased in recent years; 2017 marked the first year that more civilians were killed or injured by air-launched weaponry than any other type of weapon in the Syrian conflict [13]. An analysis of civilian deaths in Syria from 2011 to 2016 reported shelling and aerial bombardment to be responsible for 57.3% of civilian deaths [14]. MSF was a singular witness to the events in Raqqa during the offensive and post-offensive periods, and its experience indicates that the presence of active combat may not be a sufficient indicator to anticipate population need for trauma care in the context of war.

The numbers and demographics of those who were unable to access care during the two time periods described remain unknown. Barriers to timely surgical care in humanitarian settings are well known and include security, distance, lack of transport, restricted access (checkpoints) and lack of pre-hospital stabilization for critical wounds [15]. The accessibility of facilities and the scale of the medical humanitarian response differed greatly during the offensive and post-offensive period and may have had a strong impact on the number of patients that arrived at the MSF district hospital. Active fighting and restricted movement during the offensive period likely inhibited access to timely care, particularly for severe cases, meaning that patients with severe or multiple injuries perhaps did not reach care before succumbing to their injuries. In contrast, accessibility during the post-offensive period was likely easier in the absence of direct attacks, fewer checkpoints, the addition of an MSF TSP within the city limits and linked MSF ambulances. Therefore, the higher number of patients treated in the post-offensive period, as well as the higher number of patients with multiple injuries, may be the result of survival bias due to better accessibility for patients rather than a change in the burden or pattern of injury.

Both time periods showed a preponderance of male patients. During the offensive period, men remaining in Raqqa City may have been prevented from leaving urban areas, may have stayed to look after their property or may have been involved in the fighting themselves and were more exposed to injury. In the post-offensive period, MSF teams on the ground received anecdotal reports that men returned to their homes before women and children to gauge the security situation and check on their property, which may explain the higher representation of men among the blast injured in the area during that time. The disproportionate numbers of men receiving care in the Syrian conflict has been documented in other studies: Hornez et al. recorded males as 85% of cases with a median age of 27, and Arafat et al. noted 82.1% of cases were male with half of the cases falling between 19 and 35 years of age [16, 17].

Paediatric patients experience higher rates of mortality for trauma even when injury severity is similar to adults and are more prone to tertiary blast injuries; therefore, their specific medical needs should be carefully considered in conflict settings [18]. Paediatric patients were still extensively affected by the violence during the offensive and post-offensive periods. The proportion of paediatric patients seen in the offensive period (42.3%) was higher than the number of paediatric patients (24.9%) in the post-offensive period. However, this difference should be carefully interpreted, as it is possible that more children passed away from wounds before accessing care or, alternatively, that there were fewer children present in the city by the time of the post-offensive period.

The length of stay for the cohorts of blast patients seen (4 days during offensive and 7 days post offensive period) is somewhat longer than that for weapon wounded trauma patients observed by Edwards et al. in Afghanistan (mean of 3.4–4.5 days), and in Iraq (mean of 2.8–3.4 days) [19]. The longer length of stay in the post-offensive period likely reflects the complexity of cases treated, which necessitated longer inpatient time and multiple interventions for the full course of care. This workload should be considered in the planning stages of a response for blast-wounded patients to assure that the facility has capacity to manage mid to long-term cases while continuing to admit new ones.

The inpatient mortality rates for cases admitted in this series, although lower than reported in Mosul, were low as compared to other studies (Nerlander et al) and Akkucuk et al. documented a mortality rate of 14.5% while Hakimoglu et al. saw 10.4% mortality over 2 years in a cross-border facility caring for Syrian civilian wounded in Turkey, compared to the 4.1% mortality rate for blast wounded seen in the offensive period and 1.3% for blast wounded in the post-offensive period in this study [11, 20, 21]. Again, similar to in Mosul, this relatively low mortality rate may reflect a survival bias in that severely injured patients did not survive to access care [11]. In addition, despite the unknown quality of care in private facilities in the region, it is possible that some critical cases either defaulted, including discharge against medical advice, or were transferred despite MSF’s stated policy of not referring to facilities with unknown capability, reducing the inpatient mortality recorded in this study. Furthermore, the hospital had a large percentage of defaulters, particularly in the post-offensive period. It is possible that the less severe cases did not want to wait for care under the triage system in place at the MSF supported hospital or that patients sought more specialized care at private facilities that began to re-open during the post-offensive period.

As reported in other studies, wound care comprised the vast majority of interventions in both described periods. The soft tissue injuries inflicted by blasts result in high levels of tissue contamination with fragments and projectiles, as well as soft tissue necrosis, necessitating multiple individual procedures for treatment [22]. The interventions here included only those that occurred in the operating theatre and did not include the wound care that would have occurred as minor procedures in dressing change rooms or at the bedside, meaning that the burden of wound care is likely underestimated. Orthopedic surgery, including amputation, was the second most frequent category of intervention. Hariri et al. reported similar numbers of amputations among their blast wounded cohort as a percentage of all surgeries (3% compared to 3.9% for the offensive period and 3.1% for the post-offensive period described here) [23].

Given the burden of orthopaedic surgery and amputation, physiotherapy, rehabilitation and post-amputation care must be considered as part of the system of trauma care in conflict settings. Due to the high degree of contamination of open fractures in this context, MSF limited its operative orthopaedic interventions to external fixation in line with the principles of acute war surgery [24]. However, given the fracture burden seen in this series and the known rate of non-union and infection that result from these types of injuries, Raqqa governate is likely to have a significant future need for reconstructive surgery and physiotherapy [25].

The MSF supported district hospital was set up rapidly and operated under emergency conditions with a large and complex caseload. Patient care took precedence over the organization of medical records, which affected data quality and availability. While the study team initially attempted to collect more robust information on injury severity and outcomes, this documentation was not reliably recorded and had to be dropped from analysis due to poor quality. Patient charts were not tallied during the initial medical chart review due to issues with the archiving. The total number of cases seen in the facility during the time periods and the number of charts excluded from analysis due to missing information are unknown, a major limitation to the study. Documentation was weaker during the offensive period, which occurred concurrently with the ramp-up of MSF’s on-the-ground support to the district hospital. This weakness in documentation may have had an influence on the following: fewer number of included cases if they were excluded because of missing key elements; shorter length of stay if exit date was underestimated; lower numbers of interventions recorded if intervention forms were missing or not linked to patient charts. The study team took a conservative approach to inclusion of cases in order to assure a complete data set and to avoid overestimating results. In particular, the exclusion of cases that did not have complete information undeniably resulted in an underestimation of total blast cases. Interventions were only included if they were performed in the operating theatre by a surgeon. Minor procedures at the bedside or in dressing change rooms were not counted; meaning the burden of wound care is underestimated. Finally, due to weak documentation in the referral process, we were unable to link patient data from the TSP to care received at the district hospital. This link in documentation would have provided valuable insight to strength of referral pathways and the full course of treatment delivered to patients who arrived to the TSP. Future studies should seek to record patient care across referral pathways, document the severity of injury, and further investigate patient and provider experience in these types of contexts. Despite these limitations, the data put forth here offer unique insight into the medical humanitarian context during and after the Raqqa military offensive. Much of the data was collected in real time, and the entire dataset underwent a stringent cleaning and verification process. Thus, these data still present a high-quality description of the blast-injured cohort seen in a conflict and post-conflict setting.

## Conclusion

In the wake of the Raqqa offensive, the MSF supported district hospital witnessed an unforeseen second, and in fact larger, wave of blast cases as the population returned to their homes in a city filled with ERW and UXO. These events call attention to the high risk of traumatic injury to the population even after warring factions have vacated conflict zones, particularly when population movement cannot be predicted. Whether the caseload was heavier in the post-offensive period due to inaccessibility of facilities in the offensive period or due to a higher burden of blast cases remains unknown. However, given the recent experience of MSF in this context, medical humanitarian actors should be prepared for a continued and scaled up response in areas known to be highly contaminated with explosive ordnance.

## Data Availability

The datasets generated during and/or analysed during the current study are not publicly available due to MSF policy on data protection in highly insecure settings, but MSF has a managed access system for data sharing. Data are available on request in accordance with MSF’s data sharing policy. Requests for access to data should be made to data.sharing@msf.org.

## Footnotes

1 It should be noted that much of this data comes from MSF Facilities operating in the area at the time, including the one described in this report.
